# Exogenous erythropoietin administration attenuates intermittent hypoxia-induced cognitive deficits in a murine model of sleep apnea

**DOI:** 10.1186/1471-2202-13-77

**Published:** 2012-07-03

**Authors:** Ehab A Dayyat, Shelley X Zhang, Yang Wang, Zixi Jack Cheng, David Gozal

**Affiliations:** 1 Department of Pediatrics, Pritzker School of Medicine, Comer Children’s Hospital, The University of Chicago, 5721 S. Maryland Avenue, Suite K-160, Chicago, IL, MC 8000, USA; 2 Biomolecular Science Center, Burnett College of Biomedical Sciences, University of Central Florida, Orlando, FL, USA

## Abstract

**Background:**

In rodents, exposure to intermittent hypoxia (IH), a hallmark of obstructive sleep apnea (OSA), is associated with neurobehavioral impairments, increased apoptosis in the hippocampus and cortex, as well as increased oxidant stress and inflammation. Such findings are markedly attenuated in rodents exposed to sustained hypoxia 9SH) of similar magnitude. The hypoxia-sensitive gene erythropoietin (EPO) has emerged as a major endogenous neuroprotectant, and could be involved in IH-induced neuronal dysfunction.

**Methods and Results:**

IH induced only transiently increased expression of EPO mRNA in hippocampus, which was continued in (SH)-exposed mice. IH, but not SH, adversely affected two forms of spatial learning in the water maze, and increased markers of oxidative stress. However, on a standard place training task, mice treated with exogenously administered EPO displayed normal learning, and were protected from the spatial learning deficits observed in vehicle-treated (C) littermates exposed to IH. Moreover, anxiety levels were increased in IH as compared to normoxia, while no changes in anxiety emerged in EPO-treated mice. Additionally, C mice, but not EPO-treated IH-exposed mice had significantly elevated levels of NADPH oxidase expression, as well as increased MDA and 8-OHDG levels in cortical and hippocampal lysates.

**Conclusions:**

The oxidative stress responses and neurobehavioral impairments induced by IH during sleep are mediated, at least in part, by imbalances between EPO expression and increased NADPH oxidase activity, and thus pharmacological agents targeting EPO expression in CNS may provide a therapeutic strategy in sleep-disordered breathing.

## Background

Obstructive Sleep Apnea (OSA), a disorder characterized by repeated episodes of upper airway obstruction during sleep, is now recognized as a major health problem in all age groups, leading not only to significant cardiovascular and metabolic morbidity, but also to cognitive and behavioral deficits. The neurobehavioral impairments are associated with increased levels of oxidative stress and inflammatory markers, and reversible gray matter losses in neural sites contributing to cognitive function have been repeatedly described [1-5]. The episodic hypoxia-reoxygenation cycles during sleep that characterize OSA have been replicated in rodent models, and shown to elicit neurodegenerative changes, increased oxidant stress and inflammation, and impaired hippocampus-dependent learning [6-16]. However, exposures to sustained hypoxia of similar severity and duration are not associated with major cognitive deficits [3], suggesting that intrinsic differences in the presentation of the hypoxic stimulus elicit differential genomic and proteomic cellular responses that ultimately lead to divergent susceptibility.

Erythropoietin (EPO), a prototypic cytokine and hypoxia-sensitive gene, has been recently implicated in affording neuroprotection in conditions such as severe brain hypoxia or ischemia [1,17-20]. However, the time course of EPO expression during IH conditions mimicking OSA has not been specifically examined. We hypothesized that IH would elicit reduced induction of EPO expression when compared to sustained hypoxia conditions, which is not associated with significant deficits in hippocampal long-term potentiation [11]. Furthermore, we hypothesized that exogenous administration of recombinant human EPO would attenuate IH-induced NADPH oxidase mediated hippocampal oxidative stress injury and cognitive and behavioral deficits. In addition, we also tested the effect of EPO treatment on other behavioral paradigms for anxiety and depression, since such problems are frequently encountered in patients with sleep apnea, as well as with IH-exposures [21].

## Methods

### Animals

Male C57BL/6J mice (20–22 grams) were purchased from Jackson Laboratories (Bar Harbor, Maine), housed in a 12 hr light/dark cycle (lights on from 7:00 am to 7:00 pm) at a constant temperature (26 ±1°C). Mice were housed in groups of four in standard clear polycarbonate cages, and were allowed access to food and water *ad libitum*. All behavioral experiments were performed during the light period (between 9:00 am and 12:30 pm). Mice were randomly assigned to either IH, SH, or room air (RA) exposures. The experimental protocols were approved by the Institutional Animal Use and Care Committee and are in close agreement with the *Guide in the Care and Use of Animals*. All efforts were made to minimize animal suffering and to reduce the number of animals used.

In a subset of mice, treatment with recombinant human erythropoietin (rhEPO; Roche, Mannheim, Germany) was carried out for the duration of IH exposures and until all behavioral testing was completed. rhEPO was dissolved in 0.1 mol/L phosphate-buffered saline (PBS) containing 0.1% mouse serum albumin (Sigma) at a stock concentration of 2500 IU/mL. EPO-vehicle consisted of PBS containing 0.1% mouse serum albumin. rhEPO was delivered by intraperitoneal (IP) injection at a dose of 5000 IU/kg body weight in a daily fashion. EPO-vehicle was delivered by IP injection with a volume corresponding to that of rhEPO injection. This dosage has been shown to effectively cross the blood brain barrier in rodents [22-24].

### Intermittent and sustained hypoxia exposures

Animals were maintained in 4 identical commercially-designed chambers (30"x20"x20"; Oxycycler model A44XO, BioSpherix, Redfield, NY) operated under a 12 hour light–dark cycle (7:00 am-7:00 pm) for 14 days prior to behavioral testing. Oxygen concentration was continuously measured by an O_2_ analyzer, and was changed by a computerized system controlling gas outlets, as previously described [10], such as to generate stable initial oxyhemoglobin nadir values (SaO2) in the 65–72% range for SH, and alternating every 180 seconds with normoxia (SaO2 > 95%) for IH conditions. In addition, time-matched normoxic exposures (RA) were conducted. Ambient temperature was kept at 22–24°C.

### Behavioral testing

The Morris water maze was used to assess spatial reference learning and memory, as well as working memory. The maze protocol is similar to that described by Morris [25] with modifications for mice. The maze consisted of a white circular pool, 1.4m in diameter and 0.6m in height, filled to a level of 35cm with water maintained at a temperature of 21°C (Morris 1984). Pool water was made opaque by addition of 150 ml of non-toxic white tempera paint. A Plexiglas escape platform (10 cm in diameter) was positioned 1 cm below the water surface and placed at various locations throughout the pool. Extramaze cues surrounding the maze were located at fixed locations, and visible to the mice while in the maze. Maze performance was recorded by a video camera suspended above the maze and interfaced with a video tracking system (HVS Imaging, Hampton, UK).

Briefly, a standard place-training reference memory task was initiated and conducted for 6 days on mice in the water maze following exposures to 14 days of IH, SH, or RA. One day prior to place learning, mice were habituated to the water maze during a free swim. Place learning was then assessed over six consecutive days using a spaced training regimen that has been demonstrated to produce optimal learning in mice [26]. Each training session consisted of three trials separated by a 10 minute inter-trial interval (ITI). On a given daily session, each mouse was placed into the pool from 1 of 4 quasirandom start points (N, S, E or W) and allowed a maximum of 90 seconds to escape to the platform where the mice were allowed to stay for 15 sec. Mice that failed to escape were led to the platform. The position of the platform remained constant during the trials. 24 h following the final training session, the platform was removed for a probe trial to obtain measures of spatial bias. To assess the performance in the water maze, mean escape latencies and swim distance were analyzed.

### Reference memory

Retention tests were carried out 14 days after acquisition of the task. In the retention test, performance in a single session (two trials) was assessed, and the mean average performance of the two trials was calculated.

### Elevated plus maze (EPM)

The elevated plus maze (EPM) was used to assess anxiety. The basic measure is the animal preference for dark, enclosed places over bright, exposed places [27,28]. A 60 w light was placed above the apparatus and the test was video taped by an overhead camera. Mice were placed in the center of the maze facing a closed arm, and allowed to explore for 10 min in isolation. Each mouse received one videotaped trial. Mice prefer to enter into closed arms compared to open arms. Time spent in the dark area is viewed as avoidance or anxiety-like behavior. The following parameters were scored: (a) Percent time spent in open and closed arms; (b) number of entries to closed arms; (c) Time spent in the center. An arm entry was defined as the entry of all four feet into either one of the closed arm. Of note, the maze was cleaned with 30% ethanol between trials to remove any odor cues.

### Forced swimming test (FST)

Briefly, mice were individually forced to swim in an open cylindrical container (diameter 14 cm, height 20 cm), with a depth of 15 cm of water at 25 ± 1°C. The immobility time, defined as the absence of escape-oriented behaviors, was scored during 6 min, as previously described [29-31]. Each mouse was judged to be immobile when it ceased struggling, and remained floating motionless in the water, making only those movements necessary to keep its head above water. The average percentage immobility was calculated by a blinded experimenter.

### Erythropoietin and NADPH oxidase expression

qRT-PCR analysis of EPO, EPO receptor, and p47phox was performed using ABI PRISM 7500 System (Applied Biosystems, Foster City, CA). PCR Primers and Taqman probes for EPO and p47phox were purchased from ABI (Applied Biosystems). Each reaction (25μl) contained 2.5 μl reaction buffer (10x), 6 mM MgCl2, 0.2 μM dNTP, 0.6 μM each primer, 0.25 μl SureStar Taq DNA Polymerase and 2 μl cDNA dilutions. The cycling condition consisted of 1 cycle at 95°C for 10 min and 40 three-segment cycles (95°C for 30 s, 55°C for 60 s and 72°C for 30 s). Standard curves for gene of interest and housekeeping gene (β-actin) were included in each reaction. We found that the mRNA expression of β-actin was stable after IH or SH exposures. Expression values were obtained from the cycle number (Ct value) using the MX4000 software (Stratagene, La Jolla, CA). EPO, P47phox, and β-actin mRNA were performed in triplicates to determine the Ct-diff. These Ct values were averaged and the difference between the β-actin Ct (Avg) and the gene of interest Ct (Avg) was calculated (Ct-diff). The relative expression EPO and p47phox was analyzed using the 2^-ΔΔCT^ method. Quantitative results were expressed as the mean ± standard deviation (SD).

### Immunohistochemistry

Animals were deeply anesthetized and perfused intracardially with 4% phosphate-buffered paraformaldehyde. Serial sections were cut on a microtome. The free floating sections were incubated with a goat anti-mouse polyclonal EPO antibody(1:200 dilution; LS Biosciences;LS-C128821) and anti-NeuN (1:1000 dilution; Millipore, clone A60). Immunostained sections were further visualized with FITC-conjugated or rhodamine-conjugated 2nd antibody. Sections were initially assessed using a Nikon Ellipse E800 microscope, and subsequently with a confocal microscope (Leica TCS SP5). To present the expression patterns in a complete fashion, a montage of photomicrographs was assembled using Adobe Photoshop 8.0.

### Lipid peroxidation assay

MDA-586 kits (OxisResearch, Portland OR) were used to measure the relative malondialdehyde (MDA) production, a commonly used indicator of lipid peroxidation [31], in frontal brain cortex according to the manufacturer's instructions. Briefly, after anesthesia with pentobarbital (50 mg/kg intraperitoneally), mice were perfused with 0.9% saline buffer for 5 minutes and the cortex was dissected, snap frozen in liquid nitrogen, and stored at −80°C until assay the following day. Cortical and hippocampal tissues were homogenized in 20 mM phosphate buffer (pH 7.4) containing 0.5 mM butylated hydroxytoluene to prevent sample oxidation. After protein concentration measurements, equal amounts of proteins (2.0–2.5 mg protein from each sample) were used in triplicate to react with chromogenic reagents at 45°C in 500 μL buffer for 2 hours. The samples were then centrifuged and clear supernatants measured at 586 nm. The level of MDA production was then calculated with the standard curve obtained from the kit according to the manufacturer's instructions.

### 8-hydroxydeoxyguanosine (8-OHDG) tissue levels

Levels of 8-OHDG were measured in frontal brain cortex and hippocampus using a commercially available assay (Cell Biolabs, San Diego, CA). Briefly, cortical samples or 8-OHDG standards were first added to an 8-OHDG/BSA conjugate preabsorbed enzyme immunoassay plate. After a brief incubation, an anti–8-OHDG mAb was added, followed by an horseradish peroxidase-conjugated secondary antibody. The 8-OHDG content in the cortical samples was then determined by comparison with the 8-OHDG standard curve.

### Erythropoietin tissue levels

Tissue concentrations of EPO were measured in duplicate in hippocampal lysates using a commercially available ELISA assay (Quantikine cat #MEP00, R&D Systems, Inc, Minneapolis, MN). This assay was linear between 22–3,000 pg/ml using a standard calibration curve, and the intra- an inter-individual coefficients of variability were 4.6% and 8.4%, respectively.

### Primary neuronal cell cultures

Cortical neuronal cells were prepared from fetal mouse brain cortex at embryonic stage 14.5 days (E14.5). Manually dissociated brain cortical cells were plated in a Petri dish coated with poly-L-ornithine (0.015g/L) in a culture medium including neurobasal medium (Gibco), Mix B27(Gibco), L-glutamine 250uM, glutaMax 250uM, Antibiotic/Amycotic (Gibco) 1%. Half of the media in the wells were removed and replaced with fresh culture medium every 3 days. After 12 days in culture, primary neuronal cells were used for in vitro IH experiments. Cultured cortical neuronal cells were exposed to normoxia or IH respectively in a computer controlled cell incubator chamber that tightly controls O2 concentrations in the cell culture medium and in the cell culture chambers (Reming Bioinstruments, Redfield, NY). For normoxia treatment, cells were cultured under normal cell culture conditions (37°C, 95% air and 5% CO2 in a humid incubator). For IH or SH treatments, cell were treated with either alternations of 35min-5%O2/5%CO2 balance N2 followed by 25min-21% O2/5%CO2 balance N2 (IH) or exposed to 5%O2/5%CO2 balance N2 (SH) for 72 hrs in the presence or absence of pre-treatment with EPO in culture medium (1,500 pg/ml). Cells were collected, and RNA was isolated and subjected to qRT-PCR analysis for NADPH oxidase p47phox subunit expression, as delineated above.

### Data analysis

To elucidate the nature of interactions between IH, SH, and RA conditions, all data were initially analyzed by one way ANOVA. First, overall statistical significance was determined for the entire training period between the treatment groups. In addition, either two-way repeated measures ANOVA or MANOVA were used to analyze each trial block, followed by post-hoc Tukey tests. Similar statistical approaches were used to compare probe trial, reference memory, EPM and FST. For all comparisons, a p value <0.05 was considered to achieve statistical significance.

In all the experimental conditions, the data were divided into 6 blocks (containing 3 trials/day). We used a multivariate MANOVA model (SPSS software 17; Chicago) that included latency, pathlength and swim speed and two between factors: (1) Groups (four levels): RA-C, IH-C, RA-EPO, and IH-EPO (2) Condition (two levels): RA or IH. All *F* statistics are reported using Pillai’s Trace. The interaction of three different factors, i.e., time, condition and group were determined using this mixed model repeated measures MANOVA.

## Results

### EPO and NADPH oxidase expression

**Figure 1:**
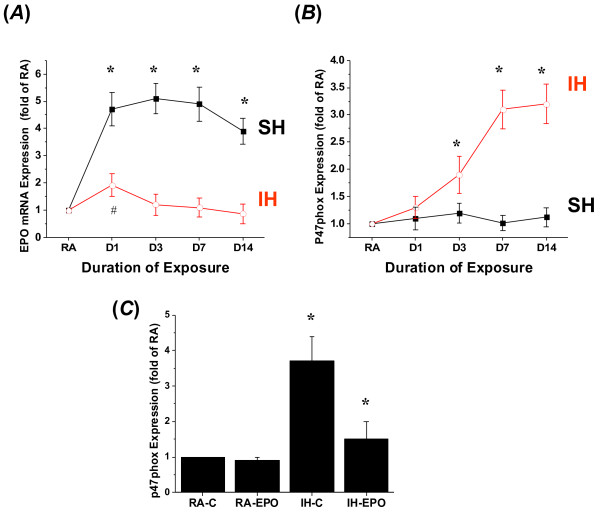
**Changes in EPO and P47phox mRNA expression in hippocampus in mice exposed to IH and SH(A)**. Changes in EPO mRNA expression in hippocampus of mice exposed to IH and SH compared to normoxic conditions (RA) (n = 7 per time point; * p < 0.01 IH vs. SH; # - IH vs. RA –p < 0.05). (**B**). Changes in NADPH oxidase P47phox subunit expression in the hippocampus of mice exposed to IH and SH (n = 7 per time point; *p < 0.01 IH vs. SH or RA). (**C**). Treatment with EPO significantly reduced p47phox subunit expression in the hippocampus of mice exposed to IH for 14 days (* - p < 0.01; n = 6 per condition). RA-C and IH-C refer to treatment with vehicle, while RA-EPO and IH-EPO indicate treatment with exogenous EPO.

Cortical tissues from IH-, SH, and RA-exposed mice were subjected to quantitative RT-PCR. Compared to normoxia (RA), EPO expression was increased after 1 day SH and heightened expression levels were sustained throughout the exposures (Figure 1A). However, although a significant, albeit attenuated increase in EPO mRNA occurred with IH, such changes were not sustained (Figure 1A). In contrast, p47 subunit of NADPH oxidase (P47phox) expression was increased in IH starting at 3 days and sustained thereafter (Figure 1B), and such changes were not apparent following SH (Figure 1B). Although no changes occurred in EPO receptor expression during IH, mild increases in mRNA expression of the EPO receptor emerged after SH at days 7 and 14 of exposures (1.8 ±0.4 and 2.1 ± 0.5 fold of normoxic controls, respectively; n = 6; p < 0.03). Furthermore, EPO treatment in IH-exposed mice resulted in significant in vivo reductions in P47phox expression (Figure 1C).

**Figure 2:**
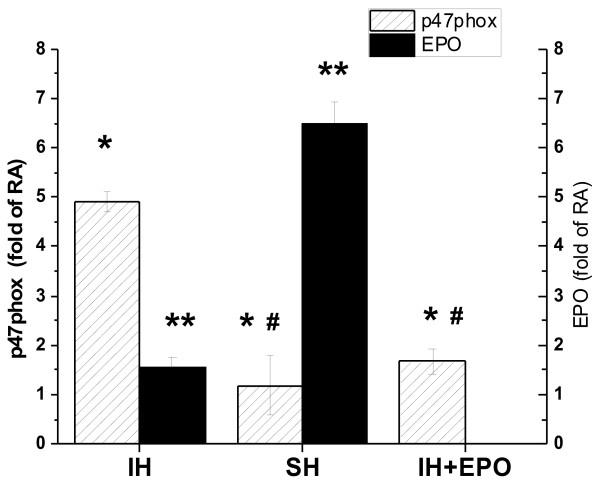
**Changes in EPO and P47phox mRNA expression in primary murine neuronal cell cultures exposed to IH, SH, and normoxic conditions (RA).** Data shown as mean ± SD of 4 separate experiments. * - p < 0.05, IH vs. SH or vs. IH + EPO; ** - p < 0.05 for EPO changes in IH vs. SH; # - p < 0.05 IH + EPO vs. SH.

In a parallel study using embrionically derived neuronal primary cells, in vitro IH for 72 hours induced significant increases in P47phox mRNA expression that were not present in SH-exposures (Figure 2). Conversely, marked increases in EPO mRNA occurred after SH, but not after IH (Figure 2). Furthermore, addition of EPO at concentrations similar to those found in brain tissue lysates during SH (i.e., 1,500 pg/ml), markedly reduced p47phox gene expression during IH (Figure 2).

**Figure 3:**
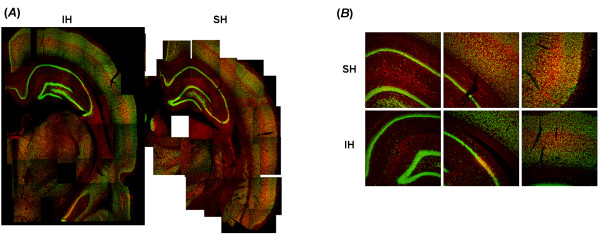
**Erythropoietin immunoreactivity in cortex and hippocampus following intermittent and sustained hypoxic exposures in mice.** (**A**). Composite reconstructions of immunohistochemically processed brain sections double labeled for EPO (red fluorescence) and NeuN (green flurorescence) immunoreactivity after IH (left) and SH (right). (**B**). Illustrative examples of EPO and NeuN labeling of hippocampus and frontal cortex after IH or SH exposures.

**Figure 4:**
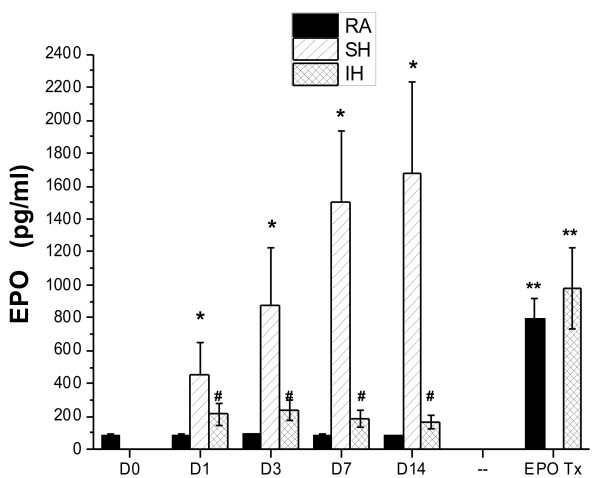
**Erythropoietin hippocampal tissue concentration changes in mice exposed to room air (normoxia, RA), IH, and SH for 1, 3, 7 and 14 days, and after administration of exogenous EPO (EPO Tx) for 14 days in RA and IH-exposed mice.** (n = 8/experimental group; * - p < 0.01 SH vs. RA or IH; # - p < 0.05 IH vs. RA; ** - p < 0.01 EPO Tx vs. RA and IH).

EPO immunoreactivity was markedly increased in SH-exposed mice, and seemed to co-localize with both NeuN-positively labeled cells and other cells. However, changes were absent or markedly attenuated in IH-exposed mice (Figure 3). To further confirm EPO changes in IH and SH conditions, EPO protein levels were also assayed in hippocampal lysates harvested from mice exposed to IH, SH or normoxic controls (n = 8/experimental group). SH was associated with marked increases in EPO tissue ocncentraitons at at all time points, and such increases, although significant when compared to normoxic conditions, were markedly attenuated in IH exposures (Figure 4). In addition, EPO administration was associated with significant increases in hippocampal EPO tissue ocncentrations (Figure 4).

### Effects of IH, SH and EPO on blood hematocrit

Normoxic mice treated with vehicle had mean hematocrits (Hct) of 34.3 ± 1.7% as compared to significant increases in Hct among EPO-treated normoxic mice (54.5 ± 3.3%; p < 0.001). SH exposures were also associated with significant and robust increases in Hct (53.1 ± 3.7%; p <0.001 vs. RA). However, IH exposures coupled with vehicle treatment did not induce significant changes in Hct (35.7 ± 1.3%; p > 0.05 vs. RA). Finally, EPO administration in IH-exposed mice induced substantial increases in Hct (56.8 ± 3.1%; p < 0.001 vs. IH + vehicle) that were similar to the effects of EPO in normoxic mice.

### Spatial learning performance

**Figure 5:**
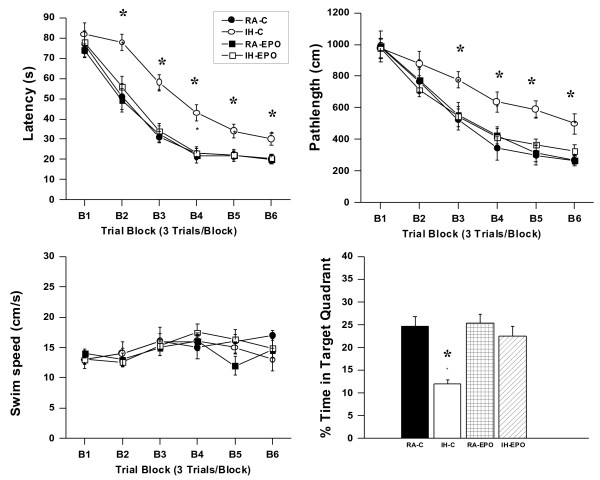
**Erythropoietin-treated mice exposed to IH do not exhibit deficits in learning and memory functions.** (**A** and **B**) Mean latencies (s) and pathlengths (cm) to locate the target platform during place training in mice either exposed to intermittent hypoxia (IH) or maintained in room air (RA) (n = 24 per group; * - p < 0.001), and receiving either vehicle (**C**) or EPO. (**C**) Swim Speed (**D**) Mean percentage time in the target quadrant during probe trial after completion of water maze testing (n = 24/experimental group;* -p < 0.001).

**Figure 6:**
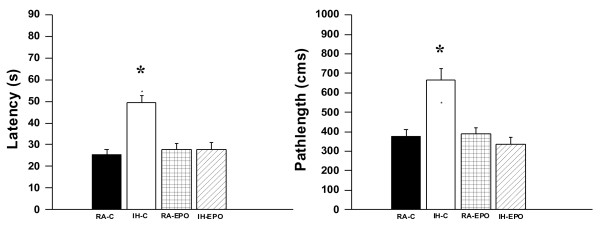
**Erythropoietin-treated mice exposed to IH do not exhibit any deficits in retention.** (**A**) Mean latencies (s) and (**B**) pathlengths (cm) to locate the target platform during retention in mice either exposed to intermittent hypoxia (IH) or maintained in room air (RA), and receiving either EPO or vehicle (C) during retention of the Morris water maze task. (n = 24/experimental group;* - p < 0.001).

On a standard place discrimination task, wild type mice exposed to 14 days of IH (IH-C) exhibited longer latencies and pathlengths to locate the hidden platform when compared to room air controls RA-C, RA-EPO, and IH-EPO mice exposed to 14 days IH (n = 24 per experimental condition; Figure 5A and B). Overall latency analysis for the entire trial blocks revealed significant changes between the different treatment groups, [F_(3,51)_ = 40.22; p < 0.001] and pathlength, [F_(3,51)_ =17.63; p < 0.001] indicating that IH adversely affected task performance in vehicle-treated mice. Significant differences in latencies were observed during blocks 2 [F_(3,51)_ =5.16; p < 0.01], 3 [F_(3,51)_ =12.43; p < 0.001], 4 [F_(3,51)_ =5.04; p < 0.01], 5 [F_(3,51)_ =10.22; p < 0.001] and 6 [F_(3,51)_ =7.67; p < 0.001]. There were no significant differences in block 1. Repeated measures ANOVA revealed significant differences in pathlengths during blocks 3 [F_(3,51)_ =7.25; p < 0.001], 4 [F_(3,51)_ =6.46; p < 0.001], 5 [F_(3,51)_ =6.58; p < 0.001] and 6 [F_(3,51)_ =5.04; p < 0.02], with no significant differences in blocks 1 and 2. There were no significant differences in swim speed in these mice. In the probe-trial test, one-way ANOVA revealed a significant effect of treatment [IH vs. RA: F_(3,51)_ =15.27; p < 0.001]. The magnitude of impairment was greatest in IH-C (Figure 5C). Of note, identical training paradigms in SH-exposed mice revealed no differences in task-acquisition performance when compared to RA-C, RA-EPO, and IH-EPO mice (data not shown). In the reference memory tests, IH-C mice exhibited significant deficits in memory retention in both latency [F_(3,51)_ =24.47; p < 0.001] and pathlength [F_(3,51)_ =19.28; p < 0.001]. However, the IH-EPO mice performed similar to RA-C (Figure 6).

Repeated measures MANOVA with latency, groups and conditions [F_(3,51)_ = 108.4; p < 0.001]; revealed that RA-C, RA-EPO, and IH-EPO treated mice as well as SH-exposed mice required significantly less time than IH-C to find the hidden platform in a Morris water maze (Figure 5); Repeated measures MANOVA with pathlength, groups and conditions [F_(3,51)_ = 42.9; p < 0.001]; indicated that as the training progressed the RA-C, RA-EPO, SH, and IH-EPO treated mice could reach the hidden platform and covered the shortest distance when compared to the distance covered by IH-C in the Morris water maze (Figure 5). In addition, repeated measures MANOVA with swim speed, groups and conditions on the swim speed showed no significant differences between the groups and treatments (Figure 5).

### Elevated plus maze and forced swim test

**Figure 7:**
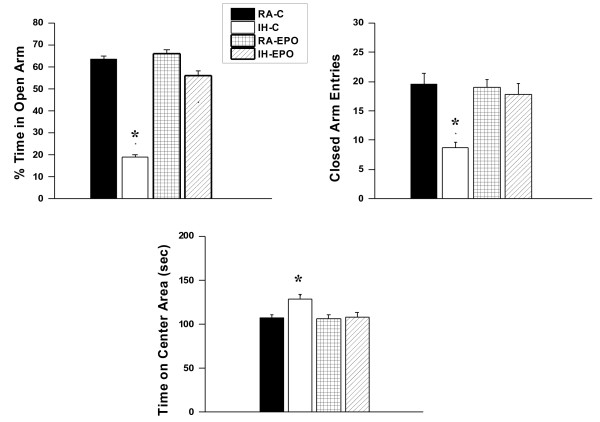
**Erythropoietin treatment prevents IH-induced anxiety in mice**. (**A**). Mice exposed to IH and treated with vehicle (IH-C) spend significantly less time in the open arm of the elevated plus maze compared to IH-EPO, RA-EPO, or RA-C mice (n = 18/experimental group;*-p < 0.001). (**B**). A reduced number of closed-arm entries emerged in mice exposed to IH and treated with vehicle (n = 18/experimental group;* - p < 0.001). (**C**). Time spend in the Center Area was increased in mice exposed to IH and treated with vehicle (n = 18/experimental group;* - p < 0.001).

**Figure 8:**
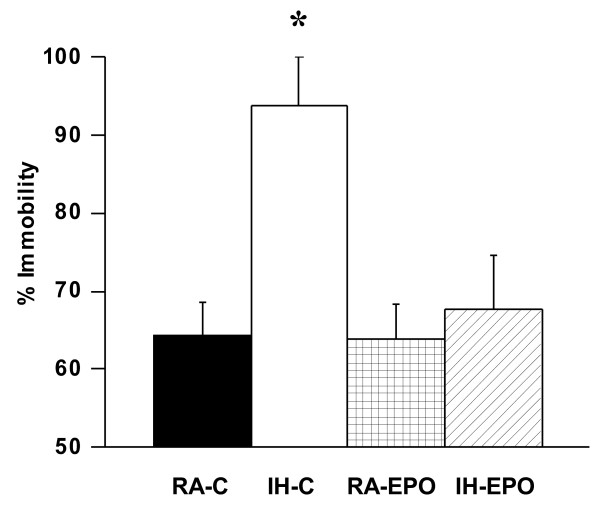
**Erythropoietin treatment prevents IH-induced increases in immobility during the forced swim test in mice.** Mice exposed to IH and treated with EPO show less immobility as compared to mice exposed to IN treated with vehicle. * p < 0.01. See text for more details (n = 18/experimental group).

IH-C mice, but not IH-EPO mice, showed significant differences in the percentage of time spent in the open arm [F_(1,48)_ = 78.21; p < 0.001] and in the number of entries into the closed arm [F_(1,48)_ = 22.67; p < 0.001] (Figure 7). The results of the elevated plus maze showed that IH-C spent significantly less time in the open arms (Figure 5; group effect, [F_(1,48)_ = 22.54; p < 0.001]) and significantly more time in the center area (Figure 5; group effect, [F_(1,48)_ = 32.66; p < 0.001]). The number of entries into the closed arms was significantly increased (Figure 7; condition effect, [F_(1,48)_ = 17.44; p < 0.001]). Although, the percentage of time spent in the open arm is commonly used as a measure of anxiety, we should also point out that the time spent on the center platform of the maze and the closed arm entries also reflect anxiety-like behaviors in mice. Similarly, the overall time spent in immobility in the forced swim test was significantly higher in IH-C treated mice, while IH-EPO mice were indistinguishable from normoxic controls (Figure 8).

### 8-OHDG levels and lipid peroxidation

**Figure 9:**
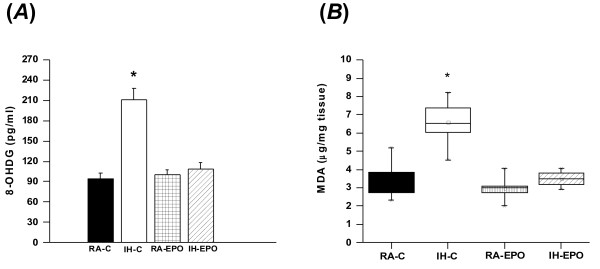
**Erythropoietin treatment reduces oxidative DNA damage and lipid peroxidation following IH exposures in mice.** 8-OHDG (**A**) hippocampal tissue levels and cortical MDA levels (**B**) in mice exposed to either room air (RA) or intermittent hypoxia and treated with either EPO or vehicle (**C**). (n = 8 per experimental group; *p < 0.001).

The levels of 8-OHDG in homogenates of cerebral cortex (data not shown) and the hippocampus were significantly higher in IH-C mice [F_(1,32)_ = 44.72; p < 0.01] and [F_(1,32)_ = 31.64; p < 0.0001] respectively; when compared to all other groups (Figure 9A). However there were no significant differences in the levels of 8-OHDG in cortex of IH-EPO when compared to either RA-C or RA-EPO controls.

Figure 9B shows MDA concentrations in homogenates of cerebral cortex from all treatment groups. A significant increase in MDA levels was observed in IH-C mice [F_(1,32)_ = 22.41; p < 0.001] in the cortex and [F_(1,32)_ = 69.13; p < 0.01] in the hippocampus when compared to all other groups.

## Discussion

OSA is a highly prevalent clinical condition across the lifespan that imposes important adverse neurobehavioral consequences. The neurocognitive and behavioral morbidity that frequently accompanies this disease stems, at least in part, from pathological inflammatory and oxidative stress processes recruited by the intermittent hypoxia that characterizes OSA [9,32]. Indeed, chronic IH has been shown to induce increased cellular levels of ROS that contribute to end-organ injury, including the CNS, a finding that is not present in SH of similar magnitude [3]. In the present study, we provide evidence that in contrast with SH exposures, episodic hypoxic events elicit only a short-lived increase in EPO expression in the brain, and conversely induce marked increases in the expression of NADPH oxidase that are absent following SH. Taken together these findings point to an imbalance between injury and defense mechanisms that is tilted towards generation of end-organ damage and dysfunction in IH. However, when EPO treatment was administered, marked reductions in lipid peroxidation and DNA oxidative damage emerged, even during IH. Consequently, cognitive and behavioral deficits associated with IH were markedly attenuated by EPO administration, suggesting that harnessing of the EPO pathway response will afford neuronal protection against the oxidative and inflammatory processes elicited by IH during sleep.

The increased expression of NADPH oxidase during IH was anticipated, and confirms previous work by Zhan and colleagues [33] and our recent work [10], showing that NADPH oxidase null mice are protected from IH-induced cognitive deficits. Of note, other ROS-generating pathways are putatively implicated in the cognitive and behavioral deficits associated with IH exposures, and reductions in oxidative stress and inflammatory signaling cascades through pharmacological interventions, and through attenuation of oxidative stress via targeted genetic manipulations of several identified target genes will all reduce or abrogate CNS dysfunction [3,9,12,13,15,34].

Depression and anxiety symptoms are frequent in OSA patients [35]. The elevated plus-maze is the most frequently utilized animal model for assessing anxiety-like behaviors [27], and provides the setting for a conflict between two innate rodent behaviors, namely avoidance of open space exposures and the tendency to explore novel environments [36]. Our current findings show that IH modified anxiety-like behavior in vehicle-treated mice exposed to IH, and that such changes in elevated maze performance disappeared upon administration of EPO, suggesting that regions underlying these behavioral responses are susceptible to IH, and the associated oxidant stress. Of note, a palliative effect on neuronal viability has been reported for apocynin, a putative NADPH oxidase antagonist in animals exposed to IH [37]. In this context, it is worthwhile to emphasize that there are multiple possible sources for oxidative stress in the context of IH [32], and it is therefore likely that such multiple sources may not only adversely affect cognitive function and EPO transcription in IH, but also that exogenous EPO administration may differentially influence the magnitude of oxidative stress in these various compartments. These issues will obviously have to await additional studies.

Considering the consensus view that assigns a coordinated role for a number of interrelated pathways, i.e., glutamate excitoxicity, oxidative stress, mitochondrial dysfunction, up-regulation of pro-inflammatory mediators, and altered regulation of pro- and anti-apoptotic gene cascades in the injurious processes associated with IH in the CNS [7,38], the protective role of EPO appears to be well justified by the previous evidence indicating that EPO is indeed operative in many of these pathways. EPO is a typical HIF-1α-dependent cytokine that has now conclusively been shown to be expressed and induced in both neuronal and glial cell populations [17]. In the last decade, multiple lines of evidence have shown that both endogenous and exogenous EPO has protective roles in CNS injury processes, such as ischemia-reperfusion injury [19,20,39-44]. Although the presence of functional EPO receptors in neurons has been challenged [45], EPO selectively reduced inflammatory and oxidative stress processes associated with brain ischemia, and prevented neuronal apoptosis [44]. Our current findings show that the modality of the hypoxic exposure is critically important for the induction of EPO expression, and that in contrast with SH, chronic IH does not result in increased EPO expression, despite similar oxyhemoglobin desaturation levels in the 2 conditions. In this context, we are unaware of specific comparisons between IH and SH and their effect on HIF-1α transcribed genes in the CNS. However, studies on other tissues such as endothelial cells [46], or perinatal adrenal chromaffin cells [38], have yielded conflicting results. Indeed, either similar or divergent HIF-1α changes have been reported. Of note, we have recently shown that SH is not accompanied by significant deficits on a spatial hippocampal task in rats [3,11], and our findings in the current study extend these observations to mice. Accordingly, systemic treatment with rhEPO, which has been shown to cross the blood brain barrier [22,23], conferred a protective effect again IH-induced oxidative stress, and prevented the cognitive and behavioral deficits associated with IH. Notwithstanding, it is possible that EPO-mediated beneficial effects may be also related to changes in angiogenesis and the cerebral microvasculature [47,48]. Indeed, evidence in children would support this assumption whereby changes in blood flow and endothelial function have been linked to cognitive function in children with sleep apnea [49,50].

## Conclusions

In summary, we have shown that prolonged SH, but not IH, induces the expression of EPO in the CNS, and the reciprocal effect occurs in the expression of NADPH oxidase during these 2 hypoxic exposures. Furthermore, we have shown that exogenous administration of EPO during the course of IH exposures mitigates the cellular oxidative stress damage and consequent behavioral impairments associated with this murine model of OSA. Although a direct mechanistic pathway can be definitively established between EPO and NADPH oxidase in the context of hypoxia-induced CNS susceptibility, this study suggests that efforts aiming to increase either EPO expression or the activation of EPO receptors in the CNS may be a promising target for OSA treatment, especially in stopping the progression, and potentially reversing the well known OSA-associated cognitive and behavioral morbidities.

## Competing interests

The authors declare that they have no competing interests.

## Authors’ contributions

ED and SW carried out the majority of the experiments, JZC performed immunohistochemistry imaging experiments, YW participated in study design and troubleshooting of some of the technical aspects of the project as well as data analysis, and DG was responsible for the conceptual framework of the project and its funding, as well as data analysis and drafting of the manuscript. All authors read and approved the final manuscript.
